# The future of radiation therapy

**DOI:** 10.1002/acm2.13141

**Published:** 2021-01-04

**Authors:** R. J. Schulz

**Affiliations:** ^1^ Yale University New Haven CT USA

Since the development of one‐ and two‐million volt x‐ray machines in the 1930s, which led to the reduction of both skin erythema and bone necrosis as well as an increase in depth dose, medical physicists have worked steadily to develop radiation sources and techniques for increasing the therapeutic ratios for most solid tumors. One of the major results of this research was the linear electron accelerator (Linac) operating in the range 4–10 MeV. Starting in the mid‐century, these machines became and remain the work‐horses of radiation therapy; they perform their tasks reliably, at acceptable cost, and with cleverly designed supplemental devices, can treat a wide range of anatomical sites.

Now, after a century of research and development, and the introduction of ever more complex and expensive radiation sources it appears that the efficacy of radiation therapy as a cancer treatment has reached a plateau. For example, proton‐beam therapy (PBT) provides treatment plans with higher therapeutic ratios, which may result in reduced radiation toxicities, but evidence that PBT offers patients longer survival times is in short supply. The following paragraph from the Mayo Clinic's website briefly summarizes the current status of PBT:

“Proton therapy has shown promise in treating several kinds of cancer. Studies have suggested that proton therapy may cause fewer side effects than traditional radiation since doctors can better control where the proton beams deposit their energy. But few studies have directly compared proton therapy radiation and X‐ray radiation, so it's not clear whether proton therapy is more effective in prolonging lives.”

Clearly, differences in survival times and levels of toxicity for PBT as compared with those for x rays are likely to be small. Therefore, to obtain statistically valid comparisons would require that relatively large numbers of patients be studied over long periods of time, and that the methodology of the randomized prospective clinical trial (RPCT) be employed. Given the logistics of running such a trial, it should come as no surprise that such trials in radiation therapy are few and far between. (Perhaps an National Cancer Institute‐sponsored, multi‐institutional RPCT of protons versus x rays for a specific cancer would help to settle this issue.) In the meantime, human nature being what it is, an oncologist who uses PBT may sincerely believe that his patients are doing better than those he used to treat with x rays, but could this not be personal bias in favor of the *new* machine whose acquisition he may have promoted for several years?

So here we are, up against what has been referred to as “The Emperor of All Maladies”^1^, a disease whose ultimate cure will likely come from biological research carried out at university and big‐pharma laboratories. While we wait, albeit impatiently, surgeons, medical oncologists, and radiation oncologists *hold the fort*, providing life‐sustaining treatments often requiring the skills of all three off these specialties. Interestingly, of the three, only the practice of radiation oncology has an inherent handicap compared with the other two. Whereas surgeons and medical oncologists can pick up a new technique or drug *overnight*, or discard one just as quickly, a new radiation source places a heavy financial burden on the hospital in the form of multi‐million dollar investment in an accelerator and a heavily shielded treatment room. Perforce, these are long‐term investments because a well‐maintained accelerator can function for generations, and a shielded treatment room, if given the time, might outlast the pyramids.

Regardless of the radiation source, 20 years from now, and short of help from synergistic drugs, radiation treatments are likely to be delivered in much the same way as they are today, and with similar results. But what is far more likely is that medical oncologists will have an increasing number of more effective drugs at their disposal, and that many of these drugs will compete with radiation therapy. This scenario accepted, it would make sense, both medically and economically, to maintain but not enlarge, our current armamentarium of several thousand Linacs and thirty‐odd PBT systems to treat the diminishing number of patients whose cancers remain refractory to drugs. As for PBT, without supporting evidence that shows significant increases in survival times, money spent on these and other new exotic radiation sources will be money taken away from far more critical general medical and hospital requirements.
